# *Fasciola hepatica* Control Practices on a Sample of Dairy Farms in Victoria, Australia

**DOI:** 10.3389/fvets.2021.669117

**Published:** 2021-06-04

**Authors:** Jane M. Kelley, Grant Rawlin, Travis Beddoe, Mark Stevenson, Terry W. Spithill

**Affiliations:** ^1^Department of Animal, Plant and Soil Sciences, Centre for AgriBioscience, La Trobe University, Bundoora, VIC, Australia; ^2^Department of Jobs Precincts and Regions, Agriculture Victoria Research, Centre for AgriBioscience, La Trobe University, Bundoora, VIC, Australia; ^3^Faculty of Veterinary and Agricultural Sciences, The University of Melbourne, Parkville, VIC, Australia

**Keywords:** *Fasciola hepatica*, triclabendazole, clorsulon, dairy cattle, survey, control strategies, diagnostic tests, farm management

## Abstract

In Australia, little is known about the strategies used by farmers to control *Fasciola hepatica* (*F. hepatica*) infection in dairy cattle. Triclabendazole-resistant *F. hepatica* have recently been found on several dairy and beef properties in Australia. It is difficult to draw conclusions about how widespread resistance is in Australian dairy cattle because we have little information about flukicide usage, drug resistance testing, and alternative flukicide usage on-farm. The study objectives were to determine how dairy farmers are currently controlling *F. hepatica* and to identify knowledge gaps where *F. hepatica* control strategies need to be communicated to farmers to improve management. The survey was distributed online or by hard copy and 36 dairy farmers completed the survey. There were 34 questions including closed, open-ended, multicheck box, demographic, and text questions. Descriptive statistics were used to quantify each response. The survey results showed high use of clorsulon, limited rotation of flukicides, and limited use of diagnostic tests to inform treatment options and timing. There was poor adherence to best management practice in determining the dose of flukicides administered to cattle, with farmers often relying on estimating body weights or average body weights, suggesting that underdosing of animals is likely to be prevalent. Most respondents in this study did not isolate and quarantine treated and newly returned or purchased animals before joining them with the main herd. The research identified four knowledge gaps where communication needs to be enhanced to improve control of *F. hepatica*: diagnostic testing to inform flukicide use, rotation of flukicide actives, flukicide administration, and increased testing of replacement animals.

## Introduction

*Fasciola hepatica* (*F. hepatica*) has been a problem in Australia since colonization (1). Early outbreaks of fasciolosis had high mortality rates and animals within irrigation regions were at higher risk (2, 3). In dairy cattle, *F. hepatica* infection reduces weight gain, milk production, and conception rates [reviewed in (4)]. Naive young cattle (calves and heifers) are more vulnerable to fasciolosis than adult stock, as they have no previous exposure to *F. hepatica* and, therefore, have no acquired immunity. Oakley et al. (5) found that *F. hepatica* infection in heifers limited growth rate, impaired feed conversion, delayed puberty, lowered conception rates, and reduced calf weight. The observed effects were more pronounced in animals that had a lower plane of nutrition.

In Victoria, replacement animals (<12 months) are isolated from adult stock in order to comply with the bovine Johne's disease program (BJD) (6). The program prevents contact between adult stock and replacements, leading to replacements being consecutively reared on the same paddocks. These paddocks tend to be more marginal and have a lower quality pasture base than grazed land provided to the milking herd. In Australia, dairy cattle predominately graze outside year-round. The key driver of the profit in pasture-based dairy farming in Australia is to increase milk produced per grazed hectare by growing more pasture of a higher quality and increasing consumption (7). Watson and Watson (8) found that the stocking rate of dairy farms across Australia has increased over the last 15 years and has reached more than two cows per hectare in some regions.

These intensive grazing strategies used on dairy farms in Australia increase pasture consumption per hectare but also increase pasture contamination with fecal matter (9). It is a growing concern that the dairy industry's intensification is increasing the development of parasite drug resistance and subclinical production losses (9). Over the last 15 years, average stocking density on dairy farms has increased from 1.51 to 1.72/ha (8). However, the stocking rate in high-intensity irrigated pasture regions of Victoria is above the national average at 1.84/ha in Loddon Valley, Torrumbarry, Central Goulburn, and Murray Valley and 2.34/ha in Macalister Irrigation District (MID). Triclabendazole (TCBZ) resistance has been confirmed on several dairy farms in these irrigated regions in Victoria (10–12). Until the early 1980s, fluke control relied on fencing off the intermediate host habitat, draining wet areas, and using flukicides of low efficacy. After the release of TCBZ, extensive work was done to communicate *F. hepatica* control strategies to farmers. Hort (13) found that 51% of sheep farmers adhered to these best practice guidelines published by the Departments of Agriculture in New South Wales and Victoria as described in Boray et al. (14). The program recommended treating in autumn (April/May) and spring (August/September) every year, with an additional summer (January) treatment for young animals and adults if they were located in high-risk regions. Since 1998, there has been no tracking of the program's adherence or effectiveness. There are only three chemical classes of flukicides registered for use in dairy cattle in Australia: TCBZ, clorsulon (CLOR), and oxyclozanide (OXY). There has also been no monitoring of how these three flukicides have been used, how diagnostic tools have been incorporated into *F. hepatica* control strategies by dairy farmers in Australia, and whether there has been an increase in the uptake of integrated parasite management strategies (IPM).

In Europe, a small number of parasite management surveys identified several knowledge gaps where *F. hepatica* control could be optimized on dairy farms (15–17). Bloemhoff et al. (15) found that 3% of dairy farmers treating for *F. hepatica* used a product unsuitable for the purpose, and grazing management options were not effectively utilized on-farm. Selemetas et al. (16) found that pasture and grazing management options had to be carefully communicated to avoid dairy farmers assuming they had a low risk of *F. hepatica* because they have good drainage. In addition, Easton et al. (17) found that there was limited use of diagnostics to inform on-farm decision-making and anthelmintic purchasing behavior was driven by factors relating to convenience. The only *F. hepatica* survey conducted in Australia was in sheep flocks and beef herds; however, only the sheep data were published (13). Hort (13) identified two knowledge gaps in how Australian sheep farmers were managing *F. hepatica*. Firstly, a high proportion of farmers were unaware that their flock was infected with *F. hepatica* because of a lack of routine diagnostic testing. The second gap was that 10% of sheep farmers used products that had no efficacy against *F. hepatica*.

The complexity of the *F. hepatica* life cycle increases the difficulty in communicating how to use flukicides, diagnostic tools, and management practices to control *F. hepatica* on-farm. It is a major hurdle in working with farmers as they need to control both the parasitic stage in cattle and snails as well as the free-living stage in waterways and on pasture, which is only possible if knowledge gaps are identified and addressed. The aim of this study was to determine the *F. hepatica* control strategies used in Victorian irrigated dairy regions. We investigated how dairy farmers control *F. hepatica*, looked for knowledge gaps in current *F. hepatica* control strategies, and identified what information needs to be communicated to farmers to improve *F. hepatica* management and reduce production losses in dairy cattle.

## Methods

### Ethical Statement

All procedures and documentation used in this study were approved by the La Trobe University Science, Health and Engineering (SHE) College Human Ethics Sub-Committee (CHESC) under negligible risk project S17-068, which was in accordance with the ethical standards outlined by the National Statement on Ethical Conduct in Human Research (2007) and the Australian Code for the Responsible Conduct of Research (2007).

### Survey Distribution and Questions

The survey was piloted in 2013 to 19 dairy farmers in the MID. After minor amendments, the survey was distributed to Victorian dairy farmers *via* hard copy and online (SurveyMonkey®) from June 1, 2017, to December 30, 2017 (Supplementary Datasheet 1). The survey consisted of 34 questions split into five sections: section 1: location and research awareness, section 2: drainage and irrigation, section 3: stock details and diagnostics, section 4: flukicides, and section 5: drenching practices. The questionnaire was made up of 16 closed questions, eight multiple choice, seven open-ended questions, two text questions, and one demographic question. No individual identifying data were collected and survey respondents were not required to complete all questions.

### Survey Respondents

Those who responded to the survey were volunteers recruited both in-person and online. The survey was advertised on the project website (www.flukecontrol.com), on dairy social media platforms, and in-person at dairy-specific events in Victoria. Respondents completed the survey during their own time. The survey allowed respondents to skip questions and provide as much or as little information as they wanted to provide. In total, 67 surveys were received and 36 respondents were included in the analysis. The authors note that recruitment took place during the “Victorian dairy crisis,” which saw large numbers of dairy farmers leave the industry, sell-off stock, and cut back on expenses and significantly lowered the confidence in the industry (18–20).

### Analysis

Online surveys were downloaded into a proprietary spreadsheet package (Microsoft Excel, Microsoft Corporation, Redmond, USA) and hard copy results were transcribed directly into the same spreadsheet. Results for the closed and multiple choice questions are presented as frequencies and percentages (%) of the total number of survey respondents. Questions that received no responses have been included in the analysis. Given the relatively small number of survey respondents, dependent variables could not be grouped by independent variable categories such as irrigation region, calving type, and herd size. Graphics were produced using Prism (GraphPad Prism version 7.03 for Windows, GraphPad Software, San Diego, California, USA, www.graphpad.com). Maps were developed using the Geographic Information System Quantum GIS (QGIS Geographic Information System; QGIS Association, http://www.qgis.org) using data obtained from the State of Victoria (21) and State of Victoria (22).

## Results

Of the 67 survey responses, 31 that were submitted online were excluded because they were incomplete (i.e., no answers were provided to any of the survey questions). In total, 36 surveys from Victorian dairy farmers were analyzed (Table 1), representing about 4.2% of the ~854 irrigated farms that are exposed to *F. hepatica* based on the known prevalence of 39% (8, 12). A response rate could not be determined as the survey was distributed online *via* email, social media, and e-newsletters as well as hard copies being handed out at industry events.

**Table 1:** A survey of *Fasciola hepatica* control practices on dairy farms in Victoria, Australia: demographic details of survey respondents.

**Question**	**Number of respondents (%)**
**Irrigation region**	
Central Goulburn (CG)	14 (39)
Macalister Irrigation District (MID)	7 (19)
Murray Valley (MV)	4 (11)
Upper Murray (UM)	4 (11)
Torrumbarry (TIA)	3 (8)
South Gippsland	2 (6)
Loddon Valley (LV)	1 (3)
Western Victoria	1 (3)
**Age (years)**	
18–24	0 (0)
25–34	7 (19)
35–44	9 (25)
45–54	12 (33)
55–64	3 (8)
65–74	3 (8)
>75	2 (6)
**Education**	
Secondary	8 (22)
TAFE or Trade qualification	4 (11)
Associate degree or diploma	10 (28)
Bachelor's degree	10 (28)
Postgraduate or master's	3 (8)
No response	1 (3)
**Gender**	
Male	26 (72)
Female	10 (28)

### Descriptive Statistics of Respondents and Their Dairy Business

**Figure 1:**
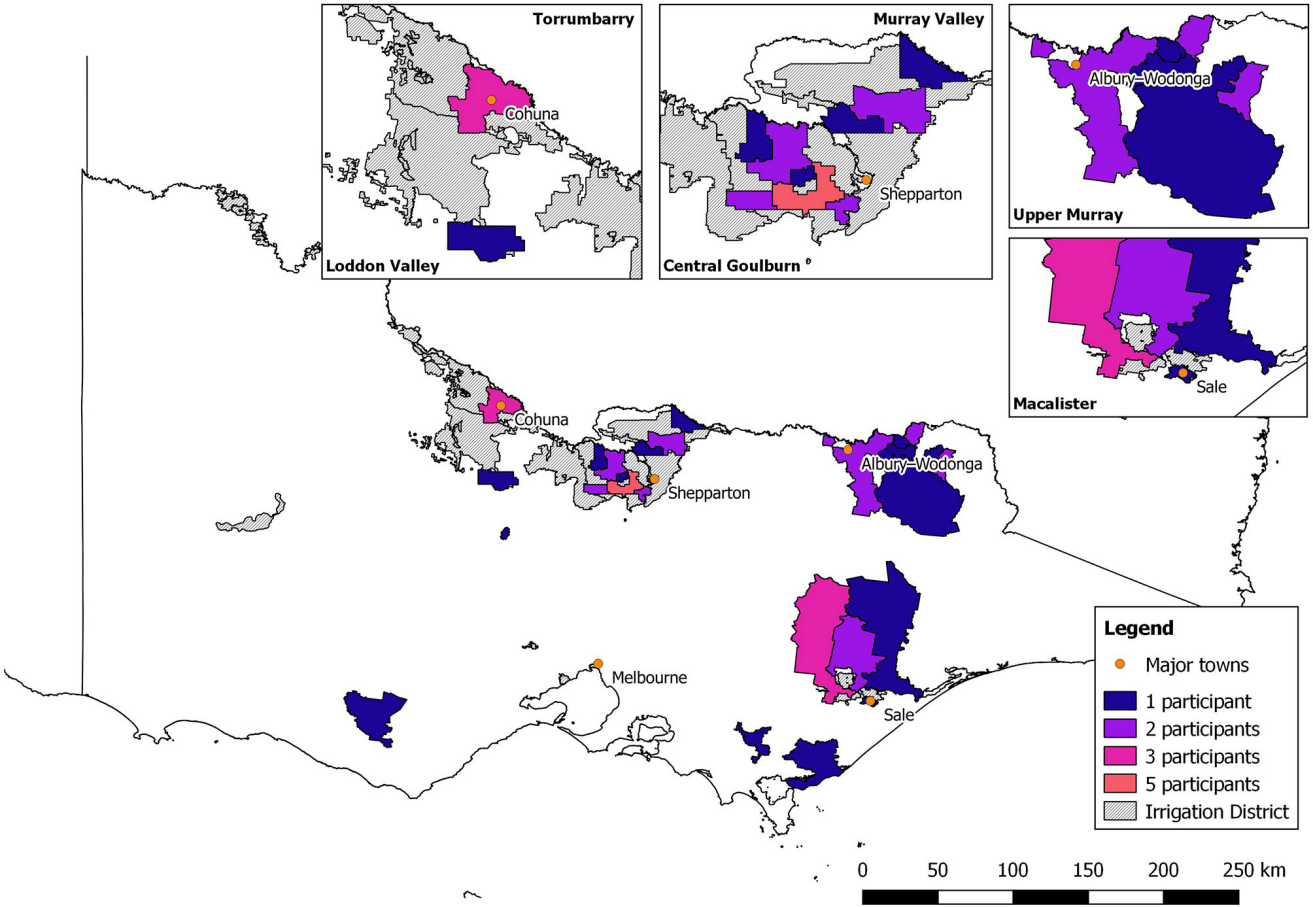
A survey of *Fasciola hepatica* control practices on dairy farms in Victoria, Australia. Map of Victoria showing the number of survey respondents by postcode area. Blue represents one respondent; purple: two respondents; pink: three respondents; and orange: five respondents. Gray hashed lines represent irrigation regions within Victoria.

Seventy-two percent of the survey respondents were male, with the majority aged between 45 and 54 years (Table 1). The highest number of surveys was received from the central Goulburn Irrigation District (*n* = 14), followed by the MID (*n* = 7). All other irrigation dairy regions were represented by at least one respondent in this study (Figure 1). The average area of all dairy farms was 427 ha, milking an average of 457 cows and rearing an average of 138 heifers and 130 calves with a total stocking density of 1.7/ha (Table 2). Of the 36 farms, 75% were split calving, 22% seasonal calving, and 3% year-round calving (Table 3). Ninety-two percent of farms had an irrigated pasture base and only one farm in the study was identified as organic (Table 3). The most frequently used method of irrigation was flood. Flood was used solely on 56% of the farms and in combination with other types of irrigation methods on 35% of the farms (Table 3). The second most common method of irrigation was center pivot, followed by laterals, sprays, and lineal move and one farm solely used a traveling gun (3%) (Table 3).

**Table 2:** A survey of *Fasciola hepatica* control practices on dairy farms in Victoria, Australia: descriptive statistics of farm area and stock numbers on each of the farms managed by the survey respondents.

**Question**	***n***	**Mean (SD)**	**Median**	**Q1, Q3**	**Min, max**
Farm area (ha)	36	427 (512)	250	150, 521	40, 2,400
No. of adults	36	457 (356)	335	249, 663	40, 2,000
No. of heifers > 12 months	36	138 (120)	120	65, 180	6, 700
No. of calves < 12 months	36	130 (102)	93	64, 203	0, 500

**Table 3:** A survey of *Fasciola hepatica* control practices on dairy farms in Victoria, Australia: types of farms, details of irrigation methods, and details of calving systems on each of the farms managed by the survey respondents.

**Question**	**Number of respondents (%)**
**Organic dairy system**	
Yes	1 (3)
No	35 (97)
**Farm type**	
Irrigated pasture base	33 (92)
Dryland pasture base	1 (3)
No response	2 (6)
**Irrigation**	
Flood	20 (56)
Traveling gun	1 (3)
Flood and center pivot	4 (11)
Flood and lineal move	1 (3)
Flood and laterals	2 (6)
Flood and spray	2 (6)
Flood, center pivot, and linear move	2 (6)
Flood, center pivot, and laterals	1 (3)
None	1 (3)
No response	2 (6)
**Calving system**	
Year-round	1 (3)
Split calving	27 (75)
Seasonal calving	8 (22)

### Dairy Farm Management

All but two survey respondents identified that their farms had problems with waterlogging (Table 4). The highest proportion (53%) reported that between 1 and 19% of their farmland had problems with waterlogging and 78% stated that stock had access to these areas (Table 4). In addition, 61% of the respondents reported that stock had access to irrigation channels on their farms. Eighty-six percent of the respondents (31/36) regularly conducted irrigation channel maintenance, often using a combination of methods to improve water use efficiency. The most common methods were spraying for weeds, fixing leaking delvers, and excavating irrigated channels (Table 4). Two respondents included other maintenance practices: one grazed channels with stock and the other replaced channels with pipes (Table 4).

**Table 4:** A survey of *Fasciola hepatica* control practices on dairy farms in Victoria, Australia: percentage of farm waterlogged at any time during the year, whether or not cattle have access to waterlogged areas, and details of irrigation maintenance on each of the farms managed by the survey respondents.

**Question**	**Number of respondents (%)**
**Percentage of farm waterlogged**	
0	2 (6)
1–19	19 (53)
20–39	3 (8)
40–59	6 (17)
60–79	2 (6)
80–99	4 (11)
100	0 (0)
**Cattle access to waterlogged areas**	
Yes	28 (78)
No	5 (14)
No response	3 (8)
**Irrigation maintenance**	
Excavate	1 (3)
Spray weeds	3 (8)
Spray weeds and excavate	3 (8)
Graze with stock and excavate channels	1 (3)
Spray weeds and fix leaking delvers	11 (31)
Spray weeds, fix leaking delvers, and excavate channels	11 (31)
Spray weeds, replace delvers with pipes, and fix leaking delvers	1 (3)
No response	4 (11)
None	1 (3)
**Access to irrigation channels?**	
Yes	22 (61)
No	11 (31)
No response	3 (8)

### *F. hepatica* Diagnostic Testing

**Figure 2:**
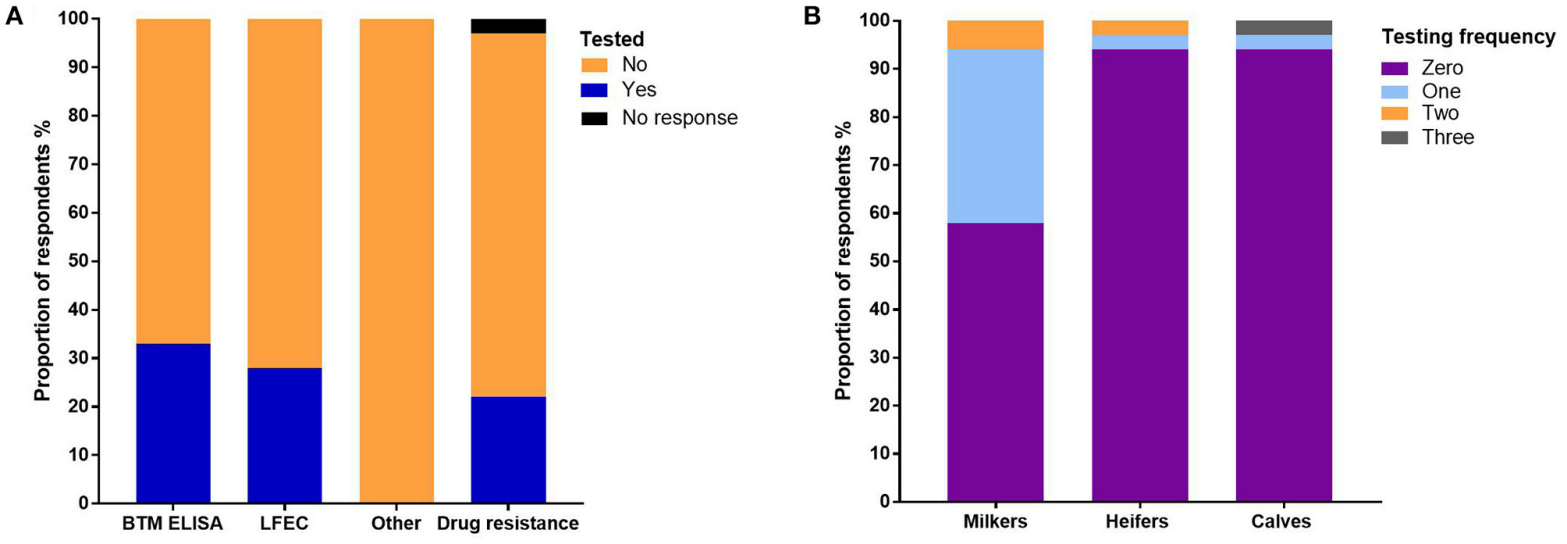
**(A)** The proportion of respondents using different types of *F. hepatica* diagnostic testing on-farm. **(B)** The proportion of respondents using various frequencies of diagnostic testing per year for each animal category.

The bulk tank milk ELISA (BTM ELISA) (23) was used to detect *F. hepatica* on 33% of farms and liver fluke fecal egg counts (LFEC) on 28% of farms (Figure 2A). No other *F. hepatica* diagnostic tests were used (Figure 2A). The highest frequency of testing occurred in adult milkers (Figure 2B). Forty-two percent of the respondents tested once per year, 6% tested twice per year, and one respondent tested three times per year (Figure 2B). For heifers and calves, only two farms tested these stock categories (Figure 2B). Nineteen percent of the respondents reported that they had tested for *F. hepatica* drug resistance, of which two stated to have worked with the lead author (Figure 2A).

### Flukicide Use

**Figure 3:**
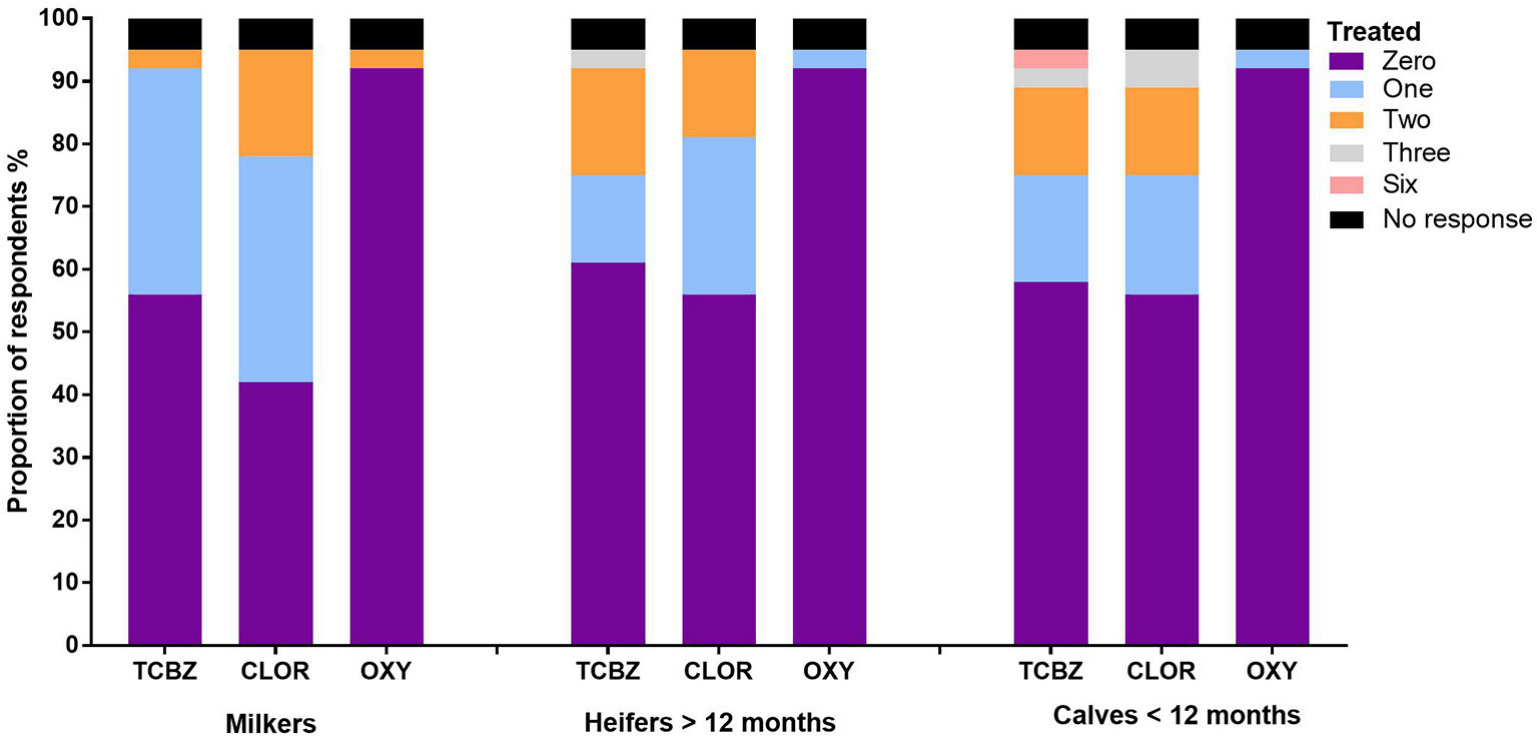
The proportion of respondents using various numbers of annual treatments with three different flukicides in each stock category (2015/2016 financial year).

**Figure 4:**
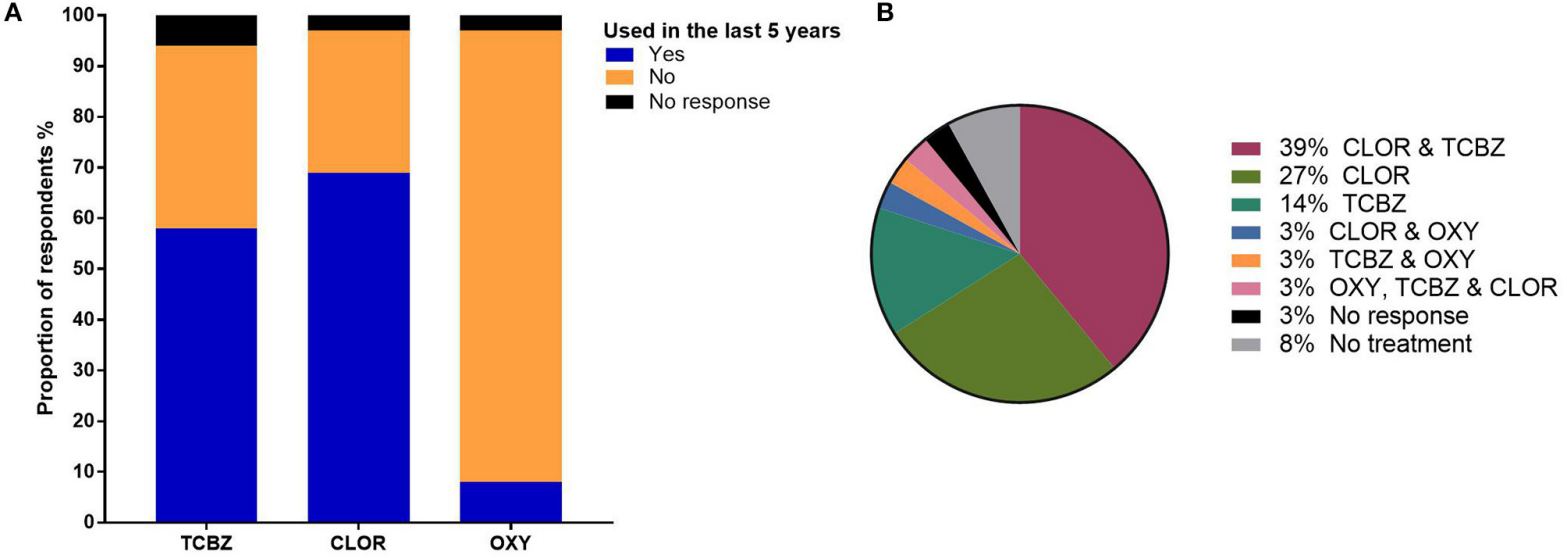
**(A)** The proportion of respondents using three different flukicides over the 5 years preceding the 2015/2016 financial year. **(B)** Proportion of respondents using single or multiple flukicides over the same time period.

**Figure 5:**
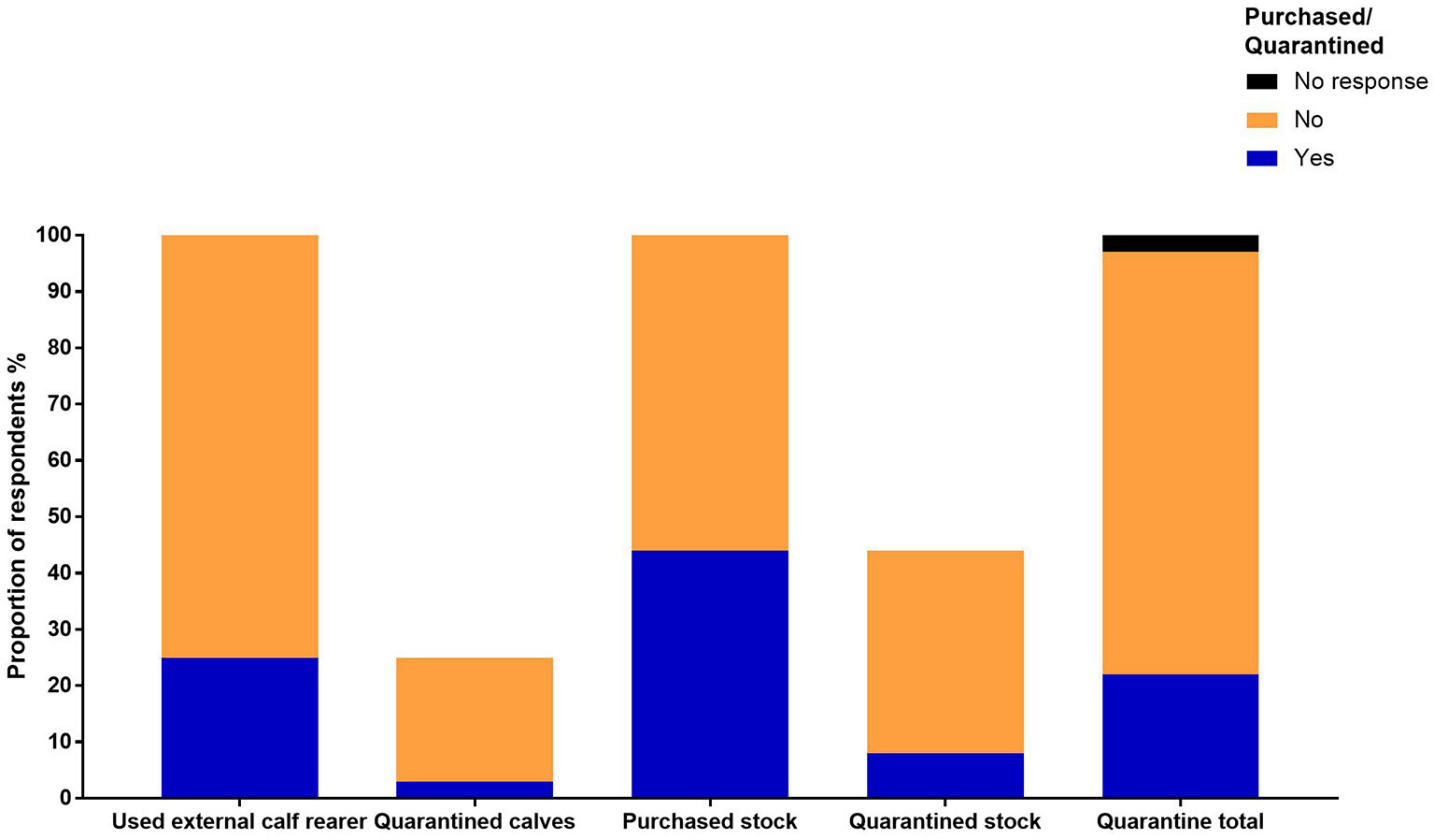
The proportion of respondents that applied quarantine treatments to calves reared or purchased externally.

In 2015–2016, 72% of the respondents treated their stock for *F. hepatica* (Table 5). TCBZ and CLOR were widely used across stock categories. The highest frequency of treatments occurred in milkers, followed by calves and heifers which received the least *F. hepatica* treatments per year (Figure 3). CLOR was most frequently used by the respondents to treat *F. hepatica*, followed by TCBZ (Figure 3). Only one respondent used OXY to treat all livestock categories (Figure 3). TCBZ and CLOR were used once or twice per year, but some opted for a higher treatment frequency in younger stock (Figure 3). The highest treatment frequency for CLOR was three times per year, whereas the highest frequency for TCBZ was six (Figure 3). For the preceding 5 years, flukicide use showed that CLOR was still the preferred product for treating *F. hepatica* in dairy cattle (Figure 4A). Several respondents used multiple flukicides to treat *F. hepatica* (Figure 4B), but 41% solely relied on one flukicide chemical class for the 5-year period. Of the respondents who reported they had either used an external calf rearer or purchased stock, only 3 and 8% of the respondents quarantine treated and newly returned or purchased animals (Figure 5).

**Table 5:** A survey of *Fasciola hepatica* control practices on dairy farms in Victoria, Australia: whether or not fluke treatment was carried out in 2015–2016 and whether or not respondents would be interested in receiving more information about fluke.

**Question**	**Number of respondents (%)**
**Treated for fluke in 2015–2016?**	
Yes	26 (72)
No	10 (28)
**More information about fluke?**	
Yes	26 (72)
No	9 (25)
No response	1 (3)

### Flukicide Administration

**Figure 6:**
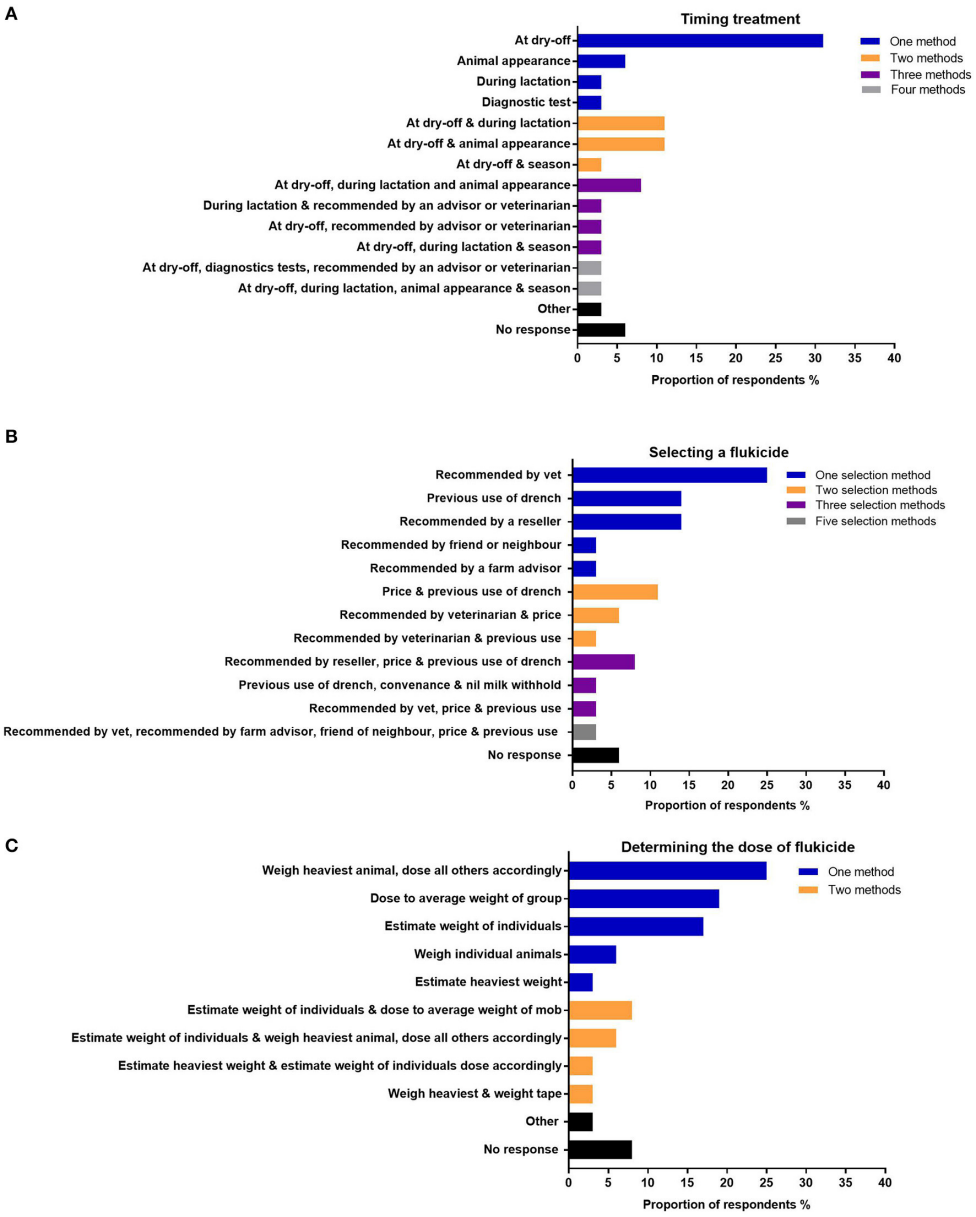
The proportion of respondents using various methods to treat animals with flukicides. **(A)** Method used to decide timing of treatment. **(B)** Method used to select a flukicide. **(C)** Method used to determine the dose of flukicide to administer to their animals.

Survey respondents used a variety of methods to determine when to treat their animals for *F. hepatica*. The most frequent approach was to treat at dry-off (31%) (Figure 6A). Other methods involved using various options: at dry-off and during lactation (11%) and at dry-off and based on animals' appearance (11%). Only two respondents used diagnostics to inform treatment administration; one respondent solely relied on diagnostics, whereas the other used it in combination with other methods (Figure 6A). Treatment based on the animal's appearance was often used to determine when to treat (Figure 6A).

When purchasing a flukicide, respondents relied more heavily on a single method of selection (59%), which was often based on advice from a veterinarian (25%), previous use (14%), or a recommendation from a reseller (14%), friend or neighbor (3%), or farm advisor (3%) (Figure 6B). Twenty-five percent of the respondents who used multiple methods to determine what flukicide to purchase often included price, previous use, and veterinarian advice as key criteria (Figure 6B).

Seventy-two percent of the respondents expressed an interest in receiving more information about *F. hepatica* drenching practices (Table 5). Sixty-nine percent of the respondents used a single method to determine the flukicide dose to be administered to their cattle (Figure 6C). A quarter of survey respondents weighed the heaviest to determine the dose for the mob, 19% used the average group body weight, 17% estimated the individual weight of animals, 6% weighed each animal, and 3% estimated the weight of the heaviest animal (Figure 6C). Nineteen percent of the respondents used a combination of methods to determine the dose; one weighed the heaviest and used a weigh tape (Figure 6C). One respondent who reported other methods in Figures 6A,C was an organic farmer who did not utilize flukicides. Instead, they incorporated copper three to four times a year into the animal's diet; the dose used was determined by a nutritionist (data not shown).

## Discussion

### Survey Response

The aim of this study was to document current fluke management practices, fluke diagnostic test use, and flukicide use on irrigated dairy farms in Victoria. Recruitment of survey respondents during the 2017 dairy crisis was difficult. The Commonwealth of Australia's Senate Economics Reference Committee (18) noted that during this time, the Australian dairy industry was facing an unprecedented crisis affecting the livelihoods of 40% of the 6,000 dairy farmers in Australia. The response rate could not be determined as the survey was distributed on multiple online platforms and hard copies were handed out at industry events. We note that three surveys were returned with a note stating the respondents had left the dairy industry. The reduced participation numbers reflect the reduced confidence in the Australian dairy industry future, which has been in decline since 2016 (75–45%) and the intention of 24% of dairy farmers to leave the industry within 5 years (19, 24). At the end of the 2015/2016 financial year, there were 4,141 dairy farms in Victoria; it has since decreased to 3,516 farms in 2018/2019 (20).

### Dairy Farms and Survey Respondents

Coverage error was present in this survey, reflected by limited geographical coverage, underrepresentation of farms in Victoria, and overrepresentation of farms and herds of larger size (Figure 1, Table 1). The Department of Agriculture and Water Resources (25) found that the average Victorian dairy farm was 252 ha, milked 345 cows, and had a stocking density of 2.1 cows/ha. The overestimation of these variables may also be a result of the phrasing of the survey question which asked for total farm area, not total usable or grazed area, which would have reduced the farms' size and increased the stocking density. Ninety-seven percent of farms had an irrigated pasture base (Table 3). The predominate method of water application was using the border-check irrigation method (known commonly as flood irrigation), which is consistent with Watson and Watson (8) and Khan et al. (26) who found 50–60% of Victorian dairy farmers solely used flood irrigation (Table 3). The descriptive statistics obtained from the 36 respondents were consistent with the work published by Schirmer et al. (27) who found the highest proportion of dairy farmers were aged between 45 and 54 years and the majority of respondents were male (>60%) (Table 1).

### Integrated Parasite Management

Non-chemical control options play a crucial role in reducing the reliance on flukicides to treat *F. hepatica*. IPM strategies focus on reducing *F. hepatica* egg contamination of pasture, restricting host access to intermediate host habitat and limiting host exposure to infective stages of *F. hepatica*. In this study, 42% of the respondents identified that more than >20% of their properties had waterlogging problems (Table 4). Host proximity to waterlogged areas, irrigation channels, and naturally occurring water bodies increases the risk of exposure and infection with *F. hepatica* (28–31). Researchers in New Zealand also identified that pugging caused by waterlogged soils increased intermediate host population (*Austropeplea tomentosa* and *Pseudosuccinea columella*) within the pasture (32). Given that in this study, stock on 78% of farms and 61% of farms had access to waterlogged areas and irrigation channels, respectively, the risk of contamination and exposure to either *F. hepatica* or the intermediate host is potentially high (Table 4). Fencing could play a key role in reducing stock access to these high-risk areas, but Watson and Watson (8) found that fencing is typically planned over a long period and is dependent on farm finances.

### *F. hepatica* Diagnostics

Our survey results suggest that we should be advocating for greater use of diagnostic tests as only 33% of farms used BTM ELISA and 28% of farms used LFEC to inform decision-making (Figure 2A). The frequency of testing was the highest in adult stock, whereas only two farms tested young animals (Figure 2B). Given that young animals are generally reared on more marginal paddocks, they are more vulnerable to *F. hepatica* and infection can have flow-on effects that impact future animal fertility, suggesting that increased testing should occur in these animals (5, 33, 34). The work by Mezo et al. (35) in Spain found that only 15% of dairy farmers tested their cattle before flukicide administration and most were unaware of the herd's *F. hepatica* status. Farmers instead relied on blanket preventative flukicide treatments. Kelley et al. (12) identified the same trend in Victorian dairy farms as several farmers were routinely treating their cattle with flukicides even though the animals were not infected with *F. hepatica*. In the United Kingdom, Easton et al. (17) found that the lowest use of diagnostic and resistance tests to inform decision-making was in the dairy industry. In this study, 19% of the respondents reported that they had tested for *F. hepatica* drug resistance (Figure 2A). Given we did not ask the farmers to explain their method for testing for resistance, it is difficult to ascertain if they followed best practice guidelines or used appropriate tests to confirm resistance.

### Flukicide Use

The survey findings suggest that the use of TCBZ and the frequency of flukicide treatments in dairy cattle have decreased from the recommendations laid out by Boray et al. (14). CLOR was more widely used in all stock categories compared with TCBZ and only one participant used OXY (Figures 3, 4A,B). The most common approach was to treat all stock categories annually except for TCBZ in heifers which were treated twice per year (Figure 3). Forty-one percent of the respondents relied on single actives (CLOR or TCBZ) and, in some cases, at a high frequency (Figures 3, 4B). Given that dairy farmers in Australia are limited to using only TCBZ, CLOR, and OXY to treat *F. hepatica*, this raises concerns about the increased selection pressures on these chemicals (Supplementary Datasheet 2) (36). A large proportion of respondents relied on CLOR, which is only sold in combination with ivermectin (Figure 3) (36). Bullen (37) found that on 15 of 20 dairy farms tested in the MID in Victoria, at least one nematode species was resistant to doramectin. Globally, there have been three reports of CLOR-resistant *F. hepatica* (38). It is challenging to assess flukicide efficacy if the product is only effective against adult *F. hepatica* (11). However, given the high use of CLOR in Australia, a methodology for testing efficacy needs to be developed. The study found that only a small number of respondents were using OXY which could be incorporated into flukicide rotations particularly in areas where TCBZ resistance has been identified in Victoria (10–12). The United Kingdom and Ireland have successfully communicated that TCBZ resistance is a growing problem, leading to increased OXY use in dairy cattle (15, 16). Another important component of IPM is to limit the introduction and spread of resistant parasites by quarantining newly purchased animals or animals returning to the farm. Most respondents in this study did not isolate and treat animals before joining them with the main herd; this breakdown in quarantine was also observed by Mezo et al. (35) on dairy farms in Spain (Figure 5).

### Flukicide Administration

Boray et al. (14) recommended treating based on the season, which only two respondents in this study used as a factor in their decision-making. Instead, most of the respondents treated at dry-off (Figure 6A). This is consistent with research in Ireland and the United Kingdom where Selemetas et al. (16) found that 96% of farmers treated at dry-off and Bloemhoff et al. (15) found that after the tightening of anthelmintic regulations, the proportion treating at dry-off increased from 59 to 81%. Only two respondents in this study used diagnostics to inform treatment timing (Figure 6A). When purchasing a flukicide, respondents relied heavily on a single selection method (59%), of which 45% selected based on advice and 14% on previous use (Figure 6B). Cornelius et al. (39) found that whoever sheep farmers sort advice from significantly influenced what other control methods were used on-farm. Farmers who relied on professionals (e.g., private veterinarians, government veterinarians, or private consultants) were more likely to use diagnostics to inform decision-making, test for resistance, drench less, and be aware of IPM. Given that veterinarians and advisors were used by many dairy farmers in selecting flukicides, one avenue for improving *F. hepatica* management would be to educate those professionals who work with dairy farmers (Figure 6B). This approach could then be extended to include rural resellers. Easton et al. (40) in the United Kingdom surveyed prescribers of anthelmintics and identified several knowledge gaps which were then addressed to improve advice given to farmers at point of purchase. Another important IPM strategy is to avoid the underdosing of cattle which limits the selection pressure for resistance. Besier and Hopkins (41) established that sheep farmers were poor estimators of live weight, leading to 85% of farmers underdosing their sheep for nematode control. Eighty-six percent of cattle farmers also underestimated live weight but by a greater margin than in sheep: 47% compared with 18% underestimation (41, 42). In this study, 50% of survey respondents estimated weight and used average weights to determine flukicide doses (Figure 6C). Underdosing is likely to be prevalent within the dairy industry, given that only 40% were weighing the heaviest animal, weighed each animal, or used weigh tapes to determine dose volume (Figure 6C).

## Conclusion

Seventy-two percent of the respondents who completed the survey wanted more information on *F. hepatica* control strategies. The evidence generated from this survey has identified several areas where *F. hepatica* management in Victoria could be optimized and has identified what IPM strategies need to be communicated to dairy farmers. Our key findings are as follows: (1) diagnostic tests are underutilized to inform flukicide timing and management of *F. hepatica* in replacement animals, (2) flukicide doses were not accurately determined and underdosing is likely to be prevalent within the dairy industry, (3) there was an overreliance on single flukicide actives and OXY was rarely used to treat *F. hepatica*, and (4) non-chemical approaches were not effectively utilized and animals had considerable access to high-risk *F. hepatica* areas on-farms. Coyne et al. (43) identified that the three biggest barriers to change on sheep farms with confirmed TCBZ resistance were overcoming habitual practices, economic feasibility, and the increased complexity in implementing IPM strategies. The best way forward for the dairy industry in Victoria would be, firstly, to do a more extensive (regionally representative) survey to establish regional differences in the management of *F. hepatica* to generate the evidence base for a tailored extension and control program. Secondly, we recommend that an economic study should be performed on the financial returns of implementing an IPM strategy on dairy farms in Victoria (44). These steps will generate the evidence base needed to encourage dairy farmers to overcome the barriers to change and implement IPM strategies on their farms.

## Data Availability Statement

The raw data supporting the conclusions of this article will be made available by the authors, without undue reservation.

## Ethics Statement

The studies involving human participants were reviewed and approved by La Trobe University Science, Health and Engineering (SHE) College Human Ethics Sub-Committee (CHESC). Written informed consent for participation was not required for this study in accordance with the national legislation and the institutional requirements.

## Author Contributions

JK and TS: conceptualization and writing — original draft preparation. JK, MS, and TS: methodology, formal analysis, and investigation. JK, TS, MS, GR, and TB: writing — review and editing. All authors contributed to the article and approved the submitted version.

## Conflict of Interest

The authors declare that the research was conducted in the absence of any commercial or financial relationships that could be construed as a potential conflict of interest.

## Supplementary Material

Supplementary Datasheet 1 A copy of the hardcopy survey that was disseminated to dairy producers in Victoria, Australia.

Supplementary Datasheet 2 Flukicide products registered for use in cattle in Australia have been listed.
