# Supplementary material for: Fasciola hepatica Control Practices on a Sample of Dairy Farms in Victoria, Australia
Source: Front Vet Sci. 2021 Jun 4;8:669117. doi: 10.3389/fvets.2021.669117 (PMC8213206; doi:10.3389/fvets.2021.669117)
Supplement: Supplementary Datasheet 1 — A copy of the hardcopy survey that was disseminated to dairy producers in Victoria, Australia. [file Data_Sheet_1.pdf]

SCIENCE, HEALTH AND ENGINEERING
School of Life Sciences, Department of Animal, Plant and Soil Sciences

Mailing address
La Trobe University
Victoria 3086 Australia

E j.kelley@latrobe.edu.au

MELBOURNE CAMPUS
Bundoora

LIVER FLUKE CONTROL ON VICTORIAN DAIRY FARMS

SURVEY DETAILS

Thank you for participating in the survey, it is made up of **34 questions**. The questions are either;

1) Fixed choice questions which require you to tick the appropriate box or boxes
2) Open ended questions which will require you to write a response

Please seek explanation from myself if you are unclear as we are more than happy to answer any questions you may have. The main investigator, **Jane Kelley, can be contacted at** j.kelley@latrobe.edu.au.

Please be as honest and as accurate as possible when completing the questions.

Kind regards,

Jane Kelley
PhD Candidate
Department of Animal, Plant and Soil Sciences
La Trobe University, AgriBio Building, 5 Ring Road, Bundoora, VIC 3083
J.Kelley@latrobe.edu.au

PLEASE TURN TO THE NEXT PAGE TO START SURVEY

SECTION 1: LOCATION AND RESEARCH AWARENESS

Q1 Please state your **Victorian** postcode below.

_____ _____ _____ _____

Q2 Please select the category that includes your age.

- ☐ 18 to 24
- ☐ 25 to 34
- ☐ 35 to 44
- ☐ 45 to 54
- ☐ 55 to 64
- ☐ 65 to 74
- ☐ 75 >
- ☐ Prefer not to state

Q3 Please select your highest level of education.

- ☐ Secondary
- ☐ TAFE or Trade qualification
- ☐ Associate degree or diploma
- ☐ Bachelor degree
- ☐ Post graduate or masters
- ☐ Prefer not to state
- ☐ Other (please specify) ________________________________

Q4 Please select your gender.

- ☐ Male
- ☐ Female
- ☐ Prefer not to state

Q5 Did you treat for liver fluke in 2016?

- ☐ Yes
- ☐ No

Q6 Have you previously worked with **Jane Kelley** from La Trobe University on the liver fluke study?

- ☐ Yes
- ☐ No

Q7 Would you like to receive more information about liver fluke drenching practices?

- ☐ Yes
- ☐ No

SECTION 2: DRAINAGE AND IRRIGATION

Q8 What is the total approximate area (ha) of your farm?

Total farm area: _______________ha

Q9 What percentage of your farm do you estimate can suffer from waterlogging?

- ☐ 100% — The entire farm can suffer from waterlogging
- ☐ 80% to 99%
- ☐ 60% to 79%
- ☐ 40% to 59%
- ☐ 20% to 39%
- ☐ 1% to 19%
- ☐ 0% — No waterlogging occurs on any part of the farm

Q10 Do cattle on your farm have access to these waterlogged areas?

- ☐ Yes
- ☐ No

Q11 Please state the approximate irrigated area (ha) for each irrigation type.

None	**Please proceed to section Q14 by turning the page.**
Flood irrigation	_______________ ha
Travelling gun	_______________ ha
Centre pivot	_______________ ha
Lineal move	_______________ ha
Other (please specify)	_______________________ _______________ ha

Q12 Please select what maintenance you carry out on your irrigation channels, delvers and drains.

- ☐ None
- ☐ Fixing leaking delvers
- ☐ Excavating channels
- ☐ Spraying weeds
- ☐ Other (please specify) ____________________________________

Q13 Do cattle on your farm have access to irrigation channels, delvers and drains?

- ☐ Yes
- ☐ No

SECTION 3: STOCK DETAILS AND DIAGNOSTICS

Q14 For each age group please state the number of stock.

Milkers ______________

Heifers >12 months ______________

Calves <12 months ______________

Q15 Please select your calving system.

- ☐ Year-round
- ☐ Split calving
- ☐ Seasonal calving
- ☐ Other (please specify) ________________________________

Q16 Have you ever used an external heifer rearer?

- ☐ Yes
- ☐ No

Q17 Did you purchase any stock in 2016?

- ☐ Yes
- ☐ No

Q18 In 2016, did you use the **milk test (ELISA)** to determine whether your milkers were infected with **liver fluke**?

- ☐ Yes
- ☐ No

Q19 In 2016, did you use **faecal egg counts (FECs)** to determine whether your stock were infected with liver fluke?

- ☐ Yes
- ☐ No

Q20 In 2016, did you use any other test to determine whether your stock were infected with liver fluke?

- ☐ Yes
- ☐ No

If yes, please specify below:

____

Q21 For each age group please state how many times your stock were tested for liver fluke in 2016.

Milkers ________

Heifer > 12 months ________

Calves < 12 months ________

SECTION 4: FLUKICIDES

Q22 For each age group please state how many times you used **Triclabendazole** (Example: Fasinex™, Flukare™) to treat for liver fluke in 2016.

Milkers ___________

Heifer > 12 months ___________

Calves < 12 months ___________

Q23 Have you used any **Triclabendazole** (Example: Fasinex™, Flukare™) product in the past 5 years (2010 to 2015)?

- ☐ Yes
- ☐ No

Q24 For each age group please state how many times you used **Clorsulon** (Example: Ivomec plus™, Virbamec Plus™, Bomectin F™) to treat for liver fluke in 2016.

Milkers ___________

Heifer > 12 months ___________

Calves < 12 months ___________

Q25 Have you used any **Clorsulon** (Example: Ivomec plus™, Virbamec Plus™, Bomectin F™) product in the past 5 years (2010 to 2015)?

- ☐ Yes
- ☐ No

Q26 For each age group please state how many times you used **Oxyclozanide** (Example: Nilzan LV™) to treat for liver fluke in 2016.

Milkers ___________

Heifer > 12 months ___________

Calves < 12 months ___________

Q27 Have you used any **Oxyclozanide** (Example: Nilzan LV™) product in the past 5 years (2010-2015)?

- ☐ Yes
- ☐ No

Q28 Did you use any other product in 2016 to treat for liver fluke? Please provide details below.

____

SECTION 5: DRENCHING PRACTICES

Q29 When do you drench for liver fluke? Select all that apply.

- ☐ At dry-off
- ☐ During lactation
- ☐ Only when recommended by a farm advisor or veterinarian
- ☐ Based on the appearance of animal or mob i.e. bottle jaw, ill thrift
- ☐ Based on diagnostic tests
- ☐ Other (please specify) ______________________________

Q30 How do you determine what drench to buy? Select all that apply.

- ☐ Recommended by veterinarian
- ☐ Recommended by reseller
- ☐ Recommended by farm advisor
- ☐ Recommended by friend or neighbour
- ☐ Price
- ☐ Previous use of drench
- ☐ Other (please specify) ______________________________

Q31 How do you determine the volume of drench to administer to your cattle? Select all that apply.

- ☐ Estimate weight of individuals and dose accordingly
- ☐ Weigh individual animals and dose accordingly
- ☐ Dose to average weight of group
- ☐ Weigh heaviest and dose all others according to this animal
- ☐ Other (please specify) ______________________________

Q32 Do you quarantine drench? i.e. do you isolate newly purchased cattle or cattle from another property and treat them for liver fluke prior to joining them with the main herd?

- ☐ Yes
- ☐ No

Q33 Have you ever tested for **liver fluke drug resistance** on your dairy farm?

- ☐ Yes
- ☐ No

Q34 If you have any additional information you feel is relevant to your property's liver fluke control program, please list below.

____

____

____

____

____

____

Thank you for taking the time to answer all the questions, your input is greatly appreciated. Your answers will help us to develop liver fluke management guidelines for dairy farms in Victoria.

PLEASE RETURN THE SURVEY IN THE PRE-ADDRESSED REPLY PAID ENVELOPE TO JANE KELLEY AT LA TROBE UNIVERSITY
