# Supplementary material for: Fasciola hepatica Control Practices on a Sample of Dairy Farms in Victoria, Australia
Source: Front Vet Sci. 2021 Jun 4;8:669117. doi: 10.3389/fvets.2021.669117 (PMC8213206; doi:10.3389/fvets.2021.669117)
Supplement: Supplementary Datasheet 2 — Flukicide products registered for use in cattle in Australia have been listed. [file Data_Sheet_2.pdf]

Product	Company	Composition	Use	Age of fluke killed	Host	Meat WHP [a]	Use in lactating cattle	Milk WHP [a]
Exifluke 240	Bayer Australia Ltd	Triclabendazole 240 g/L	Oral	All stages	Dairy & Beef	14 days	Not during lactation	Do not use less than 21 days before calving. Milk from treated cows must not be used for human consumption or supplied for processing for 96 hours (eight milking's) after calving.
Fasimec Cattle Oral	Elanco Australasia Pty Ltd	Triclabendazole 120 g/L, ivermectin 2 g/L	Oral	All stages	Dairy & Beef	21 days	Not during lactation	Do not use in lactating cows or within 28 days of calving where milk or milk products may be used for human consumption.
Fasinex 240	Elanco Australasia Pty Ltd	Triclabendazole 240 g/L	Oral	All stages	Dairy, Beef & Sheep	21 days	Not during lactation	Do not use in lactating cows where milk and milk products from treated cows may be used for human consumption.
Flukare C Plus Selenium	Virbac Australia Pty Ltd	Triclabendazole 120 g/L, selenium (as sodium selenate) 1 g/L	Oral	All stages	Dairy, Beef, Sheep & Goats	21 days	Not during lactation	Do not use in animals which are producing milk or milk products for human consumption. Do not use less than 21 days before calving, lambing or kidding in cows, ewes or does where milk or milk products from treated animals may be used for human consumption.
Flukazole C plus Selenium	Virbac Australia Pty Ltd	Triclabendazole 120 g/L, oxfendazole 45.3 g/L, selenium (as sodium selenate) 1 g/L	Oral	All stages	Dairy, Beef & Sheep	21 days	Not during lactation	Do not use in cows or sheep which are producing milk or milk products for human consumption. Do not use less than 21 days before calving in cows or lambing in ewes where milk and milk products from treated animals may be used for human consumption.
Genesis Ultra Injection	Boehringer Ingelheim Animal Health Australia Pty. Ltd.	Ivermectin 10 mg/mL, clorsulon 100 mg/mL	Injection	Adult Liver Fluke Only	Dairy & Beef	28 days	Heifers only before first mating	Do not use in dairy cattle (except replacement dairy heifers) that are producing or may in the future produce milk for human consumption or processing. Do not use in replacement dairy heifers after the first mating.
Baymec Gold Injection	Baymec Gold Injection	Ivermectin 10 g/L, clorsulon 100 g/L	Injection	Adult Liver Fluke Only	Dairy & Beef	42 days	Can be used during lactation	No WHP [a]
Ivomec Plus Antiparasitic Injection for Cattle	Boehringer Ingelheim Animal Health Australia Pty. Ltd.	Ivermectin 10 mg/mL, clorsulon 100 mg/mL	Injection	Adult Liver Fluke Only	Dairy & Beef	28 days	Can be used during lacation	No WHP [a]

Nilzan LV	Coopers Animal Health	Levamisole 64 g/L (≡ levamisole hydrochloride 75 g/L), oxyclozanide 150 g/L	Oral	Adult Liver Fluke Only	Dairy, Beef & Sheep	14 days	Can be used during lactation	No WHP [a]
Noromectin Plus Injection for Cattle	NORBROOK LABORATORIES AUSTRALIA PTY LTD	Ivermectin 10 mg/mL, clorsulon 100 mg/mL	Injection	Adult Liver Fluke Only	Dairy & Beef	28 days	Can be used during lactation	No WHP [a]
Virbamec Plus Injection for Cattle	Virbac Australia Pty Ltd	Ivermectin 10 g/L, clorsulon 100 g/L	Injection	Adult Liver Fluke Only	Dairy & Beef	28 days	Can be used during lactation	No WHP [a]

[a] WHP - Withholding period
